# The Transmission and Evolution of HIV-1 Quasispecies within One Couple: a Follow-up Study based on Next-Generation Sequencing

**DOI:** 10.1038/s41598-018-19783-3

**Published:** 2018-01-23

**Authors:** Fengjiao Yu, Yujie Wen, Jibao Wang, Yurong Gong, Kaidi Feng, Runhua Ye, Yan Jiang, Qi Zhao, Pinliang Pan, Hao Wu, Song Duan, Bin Su, Maofeng Qiu

**Affiliations:** 1 National AIDS Reference Laboratory, National Center for AIDS/STD Control and Prevention, Chinese Center for Disease Control and Prevention, Beijing, 102206 China; 2 Human Leucocyte Antigen Laboratory, Beijing Red Cross Blood Center, Beijing, 100088 China; 3 Department of AIDS/STD Control and Prevention, Dehong Prefecture Center for Disease Control and Prevention, Mangshi, 678400 Yunnan Province China; 4 Department of Epidemiology and Preventive Medicine, School of Public Health and Preventive Medicine, Monash University, Melbourne, 3004 Australia; 5 Beijing Key Laboratory for HIV/AIDS Research, Center for Infectious Diseases, Beijing You’an Hospital, Capital Medical University, Beijing, 100069 China

## Abstract

Next-generation sequencing (NGS) has been successfully used to trace HIV-1 infection. In this study, we investigated the transmission and evolution of HIV-1 quasispecies in a couple infected through heterosexual behavior. A heterosexual couple in which both partners were infected with HIV-1 was followed up for 54 months. Blood samples including whole-blood and plasma samples, were collected at various time points. After HIV-1 subtyping, NGS (Miseq platform) was used to sequence the *env* region of the HIV-1 quasispecies. Genetic distances were calculated, and phylogenetic trees were generated. We found both partners were infected with HIV-1 subtype circulating recombinant form (CRF), CRF65_cpx. The quasispecies distribution was relatively tightly clustered in the phylogenetic tree during early infection. Over time, the distribution of HIV-1 quasispecies gradually became more dispersed at 12^th^ months, with a progressive increase in gene diversity. By 37^th^ months, the sequences obtained for both partners formed different clusters in the phylogenetic tree. These results suggest that the HIV-1 contact tracing results generated by the Miseq platform may be more reliable than other conventional sequencing methods, which can provide important information about the transmission and evolution of HIV-1. Our findings may help to better target preventative interventions for promoting public health.

## Introduction

Viral quasispecies constitute a set of related variants within a virus population (e.g., from an infected patient) with similar mutations due to the rapidity and mutation-prone nature of viral replication^1^. The variability and frequency of human immunodeficiency virus type 1 (HIV-1) quasispecies provide useful information about the transmission and evolution of HIV-1 under the host immune pressure. The tracing of early HIV-1 infection is important for understanding the transmission and evolution of the virus, the course of infection in HIV-1-infected sex workers^2^, iatrogenic infections^3–5^ and for litigation in cases of deliberate infection^6,7^, which requires epidemiological investigations and molecular epidemiological evidence based on HIV-1 nucleic-acid sequencing^8^. The genetic relationship is determined by the genetic distance, and measurement of genetic distance between the nucleic-acid sequences of HIV variable region genes from the infected and suspected source of infection is an effective technique to trace the infection routes.

The direction of transmission can be deduced from the paraphyletic relationship. HIV-1 transmission results in a genetic bottleneck, as a single genetic variant is responsible for establishing infection, resulting in the paraphyly of source viruses with respect to those of the recipient. A subset of source viral sequences is more closely related to all recipient sequences than to other source sequences^7^. HIV-1 evolves rapidly under the pressure of the immune system in individuals. Over time, the similarity of HIV-1 strains between the source and the infected individual lessens, and the paraphyletic relationship becomes blurred, eventually disappearing^8–10^.

The principal laboratory methods used include the direct sequencing of nested PCR products, the sequencing of cloned PCR products^11^ and end-point limiting-dilution PCR^12^. These methods are used to deduce the transmission relationship, and the direction of transmission for the HIV-1 gene subtype or quasispecies. These methods are complex and have several disadvantages, including the generation of limited information. In recent years, next-generation sequencing (NGS) has been widely used for the deep sequencing of HIV-1 quasispecies for the detection of superinfection^13,14^ and the identification of antiretroviral drug resistance mutations at frequencies below the detection limit^15,16^. We have successfully used NGS (Illumina company, Miseq platform), which is simpler, cheaper and more practical than conventional methods, and developed the use of this technique for the tracing of HIV-1 infection in individuals thought to have become infected with the virus through sexual activity^17^.

In this study, we used NGS (Miseq platform) to study the transmission and evolution of the HIV-1 *env* region in a single HIV-1-concordant heterosexual couple over a period of 54 months, and to compare the potential difference between the results obtained from immune cells in whole-blood and plasma samples. Both members of the couple were in the early stages of infection.

## Results

### Subtypes and NGS sequencing

HIV-1 *gag* region fragments from all samples of the HIV-1-infected couple and controls were successfully amplified and then sequenced. The phylogenetic trees confirmed that the viral genotypes present in male partner (M) and his wife (F) were a circulating recombinant form (CRF), CRF65_cpx. The interpersonal genetic distance between the viruses from M and F was 0.1%, which was much smaller than that between F and six other local controls.

For F, amplification was successful for eight plasma samples and unsuccessful for one plasma sample. Amplification was successful for all seven whole-blood samples from F. Viral sequences were successfully amplified from all 6 plasma and 5 whole-blood samples from M, the first member of the couple to be infected. All the amplicons obtained were successfully sequenced by the Miseq platform. The various quasispecies sequences in each sample were counted and ranked in terms of their frequency, from high to low. Quasispecies sequences with counts of more than 50 were selected for further study (Table 1). The number of valid sequences obtained from F was 83290-180118 (mean, 131094) for plasma samples and 83329-175811 (mean, 122444) for the whole-blood samples. The plasma and whole-blood samples contained 23-322 (mean, 125), 12–190 (mean, 67) unique HIV-1 quasispecies, respectively. For M, the number of valid sequences obtained was 69949–172087 (mean, 114462) and 75115–259369 (mean, 166507) for whole-blood samples. The plasma and whole-blood samples obtained from M contained 41–311 (mean, 145) and 22–188 (mean, 98) unique HIV-1 quasispecies, respectively.

**Table 1:** Sequence and genetic distance information for HIV-1 quasispecies.

Sampling time	Estimated months since infection	Plasma specimen					Whole blood specimen				
		Specimen ID	No. of sequences	No. of unique quasispecies	Intrapersonal genetic distance(%)		Specimen ID	No. of sequences	No. of unique quasispecies	Intrapersonal genetic distance(%)	
					Average	Range				Average	Range
1	1 month	FP1	83290	51	0.7	0.0~0.9	FW1	106443	12	0.6	0.0~0.6
2	4 months	FP2	180118	35	0.9	0.0~1.3	FW2	125743	18	0.6	0.0~1.6
3	7 months	FP3	150266	103	1.6	0.0~2.6	FW3	171124	31	0.6	0.0~1.2
4	12 months	FP4	136249	310	1.7	0.0~3.6	FW4	150694	60	0.6	0.0~2.9
5	15 months	FP5	135538	48	0.6	0.0~1.2	—	—	—	—	—
6	18 months	FP6	90088	23	0.6	0.0~0.6	—	—	—	—	—
7	28 months	FP7	160427	111	1.7	0.0~3.8	FW7	83329	91	2.9	0.0~5.4
8	37 months	FP8	112776	322	2.3	0.0~4.8	FW8	97328	190	3.1	0.0~6.1
9	54 months	※FP9	NA	NA	NA	NA	FW9	175811	74	0.6	0.0~1.2
1	1 month	MP1	172087	41	0.6	0.0~1.5	MW1	134179	70	0.8	0.0~4.3
2	4 months	MP2	69949	129	1	0.0~2.0	MW2	259369	87	0.6	0.0~0.9
3	7 months	MP3	106222	311	1.6	0.0~3.2	MW3	211341	22	0.8	0.0~1.9
4	12 months	MP4	103859	296	2	0.0~4.5	MW4	152531	188	1.2	0.0~2.5
5	15 months	MP5	78413	51	1.2	0.0~1.9	—	—	—	—	—
6	37 months	MP8	156241	44	0.7	0.0~0.7	MW8	75115	123	2.7	0.0~5.4

The samples from F at different sampling time is numbered as FP(x), FW(x) while samples from M at different sampling time is numbered as MP(x), MW(x). “(x)” means the times of sampling. ※Subject of FP9 had been treated with antiretroviral therapy for 16 months before sampling, and the others were not treated. “—” means absent of samples. “NA” means “Not available”.

### Evolution of HIV-1 quasispecies overtime

**Figure 1:**
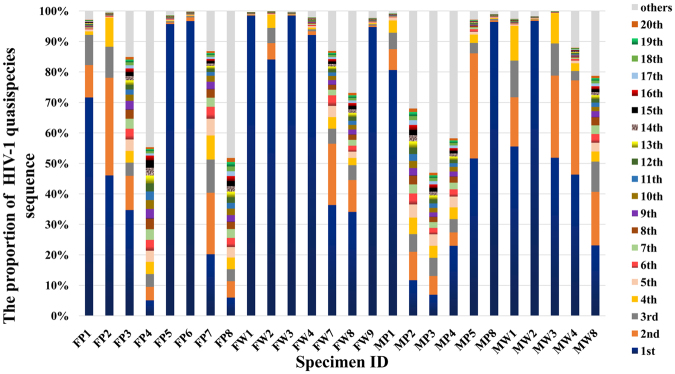
Frequency of each HIV-1 quasispecies sequence. The frequency of each quasispecies sequence in each sample was determined by counting, and ranked from high to low. Different quasispecies are indicated with different colors.

The dominant quasispecies frequency accounted for more than 70% of all sequences at the first time point, except that in whole-blood samples from M (Fig. 1). In plasma samples from F, the number of quasispecies gradually increased from the first to the fourth time point. However, the distribution of quasispecies was very concentrated at the 5^th^ and 6^th^ time points, with a gradual dispersal observed thereafter (Fig. 1). Whole-blood samples from F and plasma and whole-blood samples from M also followed similar patterns. From 1 to 37 months post-infection, the intraspecimen gene diversity gradually increased in both plasma and whole-blood samples from both M and F. For each subject, diversity between the follow-up sample and the first sample tended to increase over time (Fig. 1). Greater sequence dispersal was observed for plasma than for whole-blood samples.

**Figure 2:**
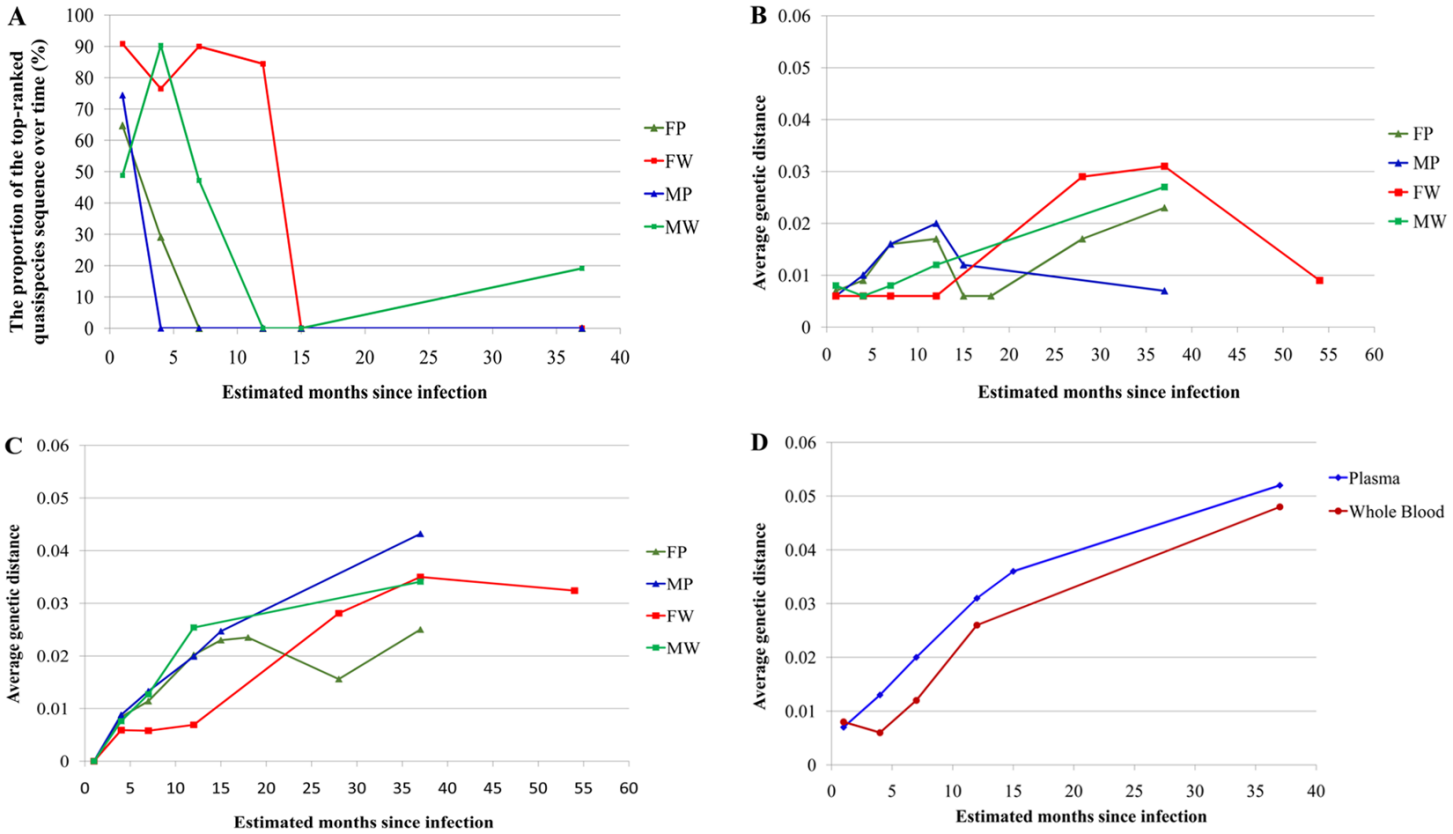
Changes in the proportion of the top-ranked quasispecies sequence over time and the influence of the infection duration on genetic distance. (**A**) The sequence is defined as that ranked 1 in the first sample. (**B**) The intraspecimen average genetic distance at different follow-up times, for whole-blood and plasma samples. (**C**) Is the average genetic distance between the first sample and follow-up samples taken at different times thereafter, for whole-blood and plasma samples. (**D**) Is the average genetic distance between the viruses present in the two partners. Blood samples including Whole-blood (MW, FW) and Plasma (MP, FP) samples, were collected from the male (M) and female (F) partners at various time points. Genetic distances were calculated with the Jukes-Cantor model in MEGA 6.0.6 software.

At baseline, other than for the whole-blood sample from M (MW), the most frequent quasispecies sequence accounted for more than 50% of the sequences obtained. As the duration of the infection increased, the distribution of the quasispecies populations changed. The overall proportion of the most frequent quasispecies decreased first, but this proportion increased in MW samples from the 15^th^ week (Fig. 2A).

### The effect of infection time on genetic distance

The genetic distance between the populations of HIV-1 quasispecies gradually increased over time in the absence of antiretroviral treatment. From the 1^st^ to the 8^th^ sample, average genetic distance in F increased from 0.7% in plasma and 0.6% in whole blood to 2.3% and 3.1%, respectively. In M, from the 1^st^ to the 6^th^ sample, average genetic distance increased from 0.6% in plasma and 0.8% in whole blood to 0.7% in plasma and 2.7% in whole blood. After 16 months of antiretroviral therapy (corresponding to a time point 54 months after the start of infection), PCR on plasma from F failed, whereas that on whole-blood samples succeeded, with an average genetic difference between HIV-1 quasispecies of 0.6%. This finding suggests that long-term antiretroviral treatment has a significant effect on the composition of the population of HIV-1 quasispecies (Fig. 2B).

Average genetic distance between the viruses in the follow-up samples and those in the first sample also tended to increase over time. This increase was slightly more marked for plasma than for whole blood in M (Fig. 2C). No amplification was achieved with the FP9 sample for 54^th^ months (Fig. 2C). The average genetic distance between the viruses present in the male and female partners increased from 0.7% in both plasma and whole blood in the first month to 5.4% in plasma and 4.7% in whole blood in the 37^th^ month after infection. Figure 2D also illustrates the average genetic distance to the first sample at different follow-up times. Overall, this genetic distance was slightly higher for plasma than for whole-blood samples.

### The influence of infection time on the inference of evolutionary mechanism from the phylogenetic tree

**Figure 3:**
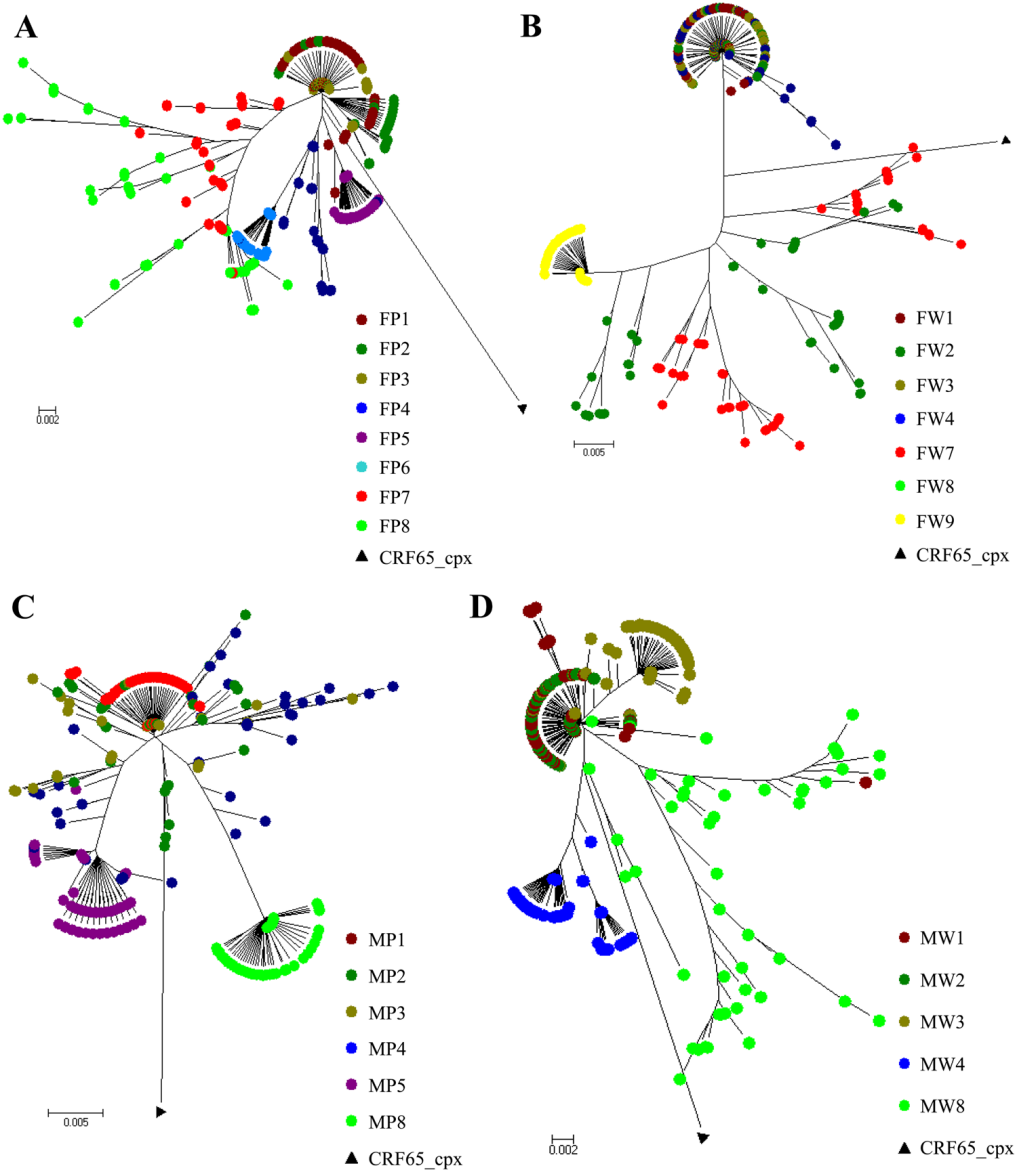
Evolution of HIV-1 quasispecies within one couple at different follow-up times from the phylogenetic tree. (**A**–**D**) The evolution of FP/MP and FW/MW. FP/MP mean the plasma samples of F and M, respectively. FW/MW mean the whole-blood samples of F and M, which were collected from the female (F) and the male (M) partners at various time points. The calculation time required was reduced by including only quasispecies identified more than 50 times.

**Figure 4:**
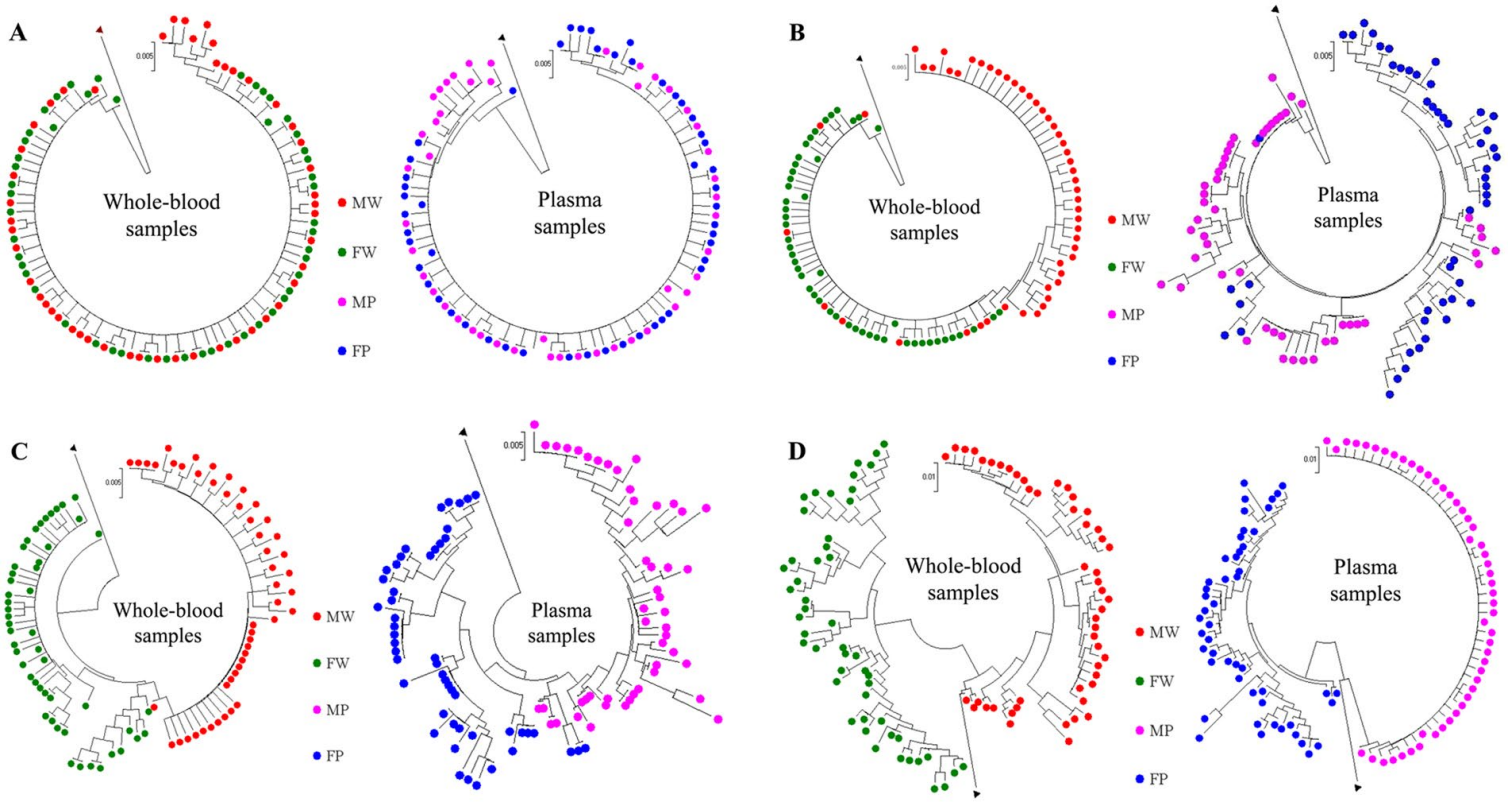
Influence of the duration of infection on the inference of the evolutionary mechanism by phylogeny. Neighbor-joining tree for quasispecies of the HIV *env* region (HXB2 position 7169 to 7520) from the male (MWx and MPx) and female (FWx and FPx) partners of the couple, based on analyses of plasma samples and whole-blood at various time points during follow-up. (**A**,**B**,**C** and **D**) correspond to 1, 7, 12, 37 months post-infection, respectively.

HIV-1 transmission results in a genetic bottleneck through sexual activity, as a single genetic variant is responsible for establishing infection, resulting in the paraphyly of source viruses with respect to those of the recipient. During acute infection HIV-1 in M has been very quickly transmitted to F. Thus, phylogenetic analyses showed that the sequences from M and F clustered together, but it was difficult to identify the direction of transmission (Figs 3 and 4). The distribution of quasispecies population was fairly narrow in early infection with a close genetic distance, leading to tight clustering on the phylogenetic tree (Fig. 3A–D). However, this distribution gradually became more dispersed over time, resulting in a scattering of samples on the phylogenetic tree. In addition, analysis of the FW9 sample showed a concentration of the distribution after 16 months of antiviral therapy (Fig. 3B). Even though, the population of quasispecies present in early infection included the quasispecies predominating later in infection, no re-infection or viral recombination has been observed over time within this couple in the study (Figs 3 and 4).

Analyses based on the analysis of the phylogenetic tree obtained at 12^th^ months resulted in correct inference of the evolutionary mechanism from data for plasma, whole-blood or mixed samples. The MP3 population was located at the root of the phylogenetic tree, giving rise to a large number of MP3 quasispecies, and a small number of FP3 quasispecies, MW3 and FW3 were paraphyly-relevant, and MW3 was located at the tip of the phylogenetic tree (Figs 3 and 4). On the phylogenetic tree obtained 37 months after infection, the distance between the populations obtained from F and M was greater on this tree.

## Discussion

We used a recently developed approach based on NGS (Miseq platform) to analyze the HIV-1 quasispecies present in samples, to study the transmission and evolution of HIV-1 under the host immune pressure, and to provide molecular epidemiological data for the tracing of HIV-1 infection. We found that HIV-1 quasispecies richness tended to increase early in infection, but that the diversity of the quasispecies population was low at specific time points (FP5, FP6, FW9, MP8, MW2). The virus may have escaped host immunity through rapid evolution, in which case quasispecies richness might be expected to continue to increase during early infection. Viral adaptation to the host environment resulted in a stable population of quasispecies, with a decrease in quasispecies richness and gene diversity. At this time point, quasispecies richness was high for all the samples except for the whole-blood sample from F. The two participants in this study had recently become infected and chose not to take antiretroviral drugs in the first 37 months post-infection. This situation provided an ideal opportunity for studying the host-virus relationship. However, given the small number of individuals studied, additional investigations will be required to determine whether the trends observed are universal and to strengthen our findings.

There is strong evidence to support the existence of a bottleneck following transmission by heterosexual intercourse. Thus, despite the diversity of the viruses present in the person transmitting the infection, only one or a few quasispecies seem to be transmitted. As shown in the Figs 3 and 4, M was infected within one month after non-marital heterosexual exposure, and then quickly transmitted viruses from M to F during acute HIV-1 infection. Thus, the quasispecies population distribution was fairly narrow in early infection with a close genetic distance, leading to tight clustering on the phylogenetic tree (Fig. 3A–D). Moreover, HIV-1 evolves rapidly in individuals, this distribution gradually became more dispersed over time, resulting in a scattering of samples on the phylogenetic tree. HIV-1 intra-patient quasispecies clustering of both partners intersect each other, and viral strains evolved independently, thus it is difficult to infer the direction of transmission. However, neither viral recombination nor intrapersonal HIV-1 transmission over time were observed for both partners in our study (Fig. 4), suggesting that the quasispecies distribution was relatively tightly clustered during very early infection, with the sequences found located within a single cluster in the phylogenetic tree (Figs 3 and 4).

The relationship and direction of transmission have been successfully inferred by genetic distance and paraphyly relevance studies in a number of cases, in infection trace ability investigations^8,10,18,19^. The duration of HIV-1 infection has a direct impact on the trace ability results. The longer the time since infection, the greater the difference between quasispecies populations is likely to be between the person acting as the source of the infection and the person infected. However, few studies have monitored HIV-1 variants. In this study, we found that the average genetic distance between the first same and follow-up samples tended to increase over time, although follow-up was cut short by the death of M in this study. These findings are consistent with the view put forward by Kupfer^20^. FW9/FP9 were the only samples obtained after the initiation of treatment, and the lack of HIV-1 DNA amplification from the plasma sample demonstrates the efficacy of the antiretroviral treatment. For whole blood, average genetic distance with respect to the first sample had increased. Overall, the average genetic distance between the viruses present in the two partners increased over time, and this difference was slightly greater for plasma than for whole blood. It remains unclear how long individuals should be followed up.

Attempts to determine the direction of transmission must take several factors into account: (1) the timing of sample collection relative to the onset of infection: the shorter the duration of infection, the easier the interpretation. The accuracy achievable at different time intervals has yet to be studied; (2) the interval between the infection events in the two individuals. Determination is easier for longer intervals. However, care is required when trying to infer the direction of transmission early in infection, particularly for longer sampling intervals.

HIV-1 superinfection may also influence evolutionary analysis. In superinfection, a previously infected individual is re-infected with another strain different from that responsible for the first infection. HIV-1 superinfection has been reported to have a worldwide incidence of 0–7.7% per year^21^. Many superinfections may go undetected due to the low sensitivity of conventional methods^21^. If HIV-1-infected couples, in addition to having sex with each other, also have high-risk extramarital behavior, then HIV-1 quasisequence analysis needs to take into account not only the two partners in the couple, but also more distant external associations. The detection of HIV-1 superinfection requires cloning or NGS techniques. Several studies on HIV-1 superinfection have been published^22–25^. For example, Redd *et al*., detected HIV-1 superinfection in couples and in female sex workers^26^ in Ugandan. When identified, cases of superinfection should be subject to additional follow-up.

In summary, the implementation of high-throughput NGS using the Miseq platform can improve the investigation of HIV transmission and subsequently intrapersonal HIV evolutionary studies. This allowed us to study the characteristics of HIV paraphyly for different HIV transmission modes under the pressure of the host immune system, in addition to the molecular epidemiologic relationship between the individuals.

## Methods

### Ethics statement

The participants provided written informed consent for their information, and clinical samples were stored and used for research. Ethical approval for this study was obtained from the institutional review board of the National Center for AIDS/STD Control and Prevention of the Chinese Center for Disease Control and Prevention, and written informed consent was provided according to the declaration of Helsinki. The methods were carried out in accordance with approved guidelines and regulations.

### Epidemiological Materials

The 46-year-old male partner (M), was found HIV-1 seroconversion within one month after non-marital heterosexual exposure, with a viral load of 110,000 copies/ml. His wife (F), who was 32 years old, was also found HIV-1 seroconversion 4 days later, with a viral load of 17,000 copies/ml. She had not engaged in any high-risk behavior other than unprotected sex with her husband. Both M and F were identified in early infection and were followed for 37 and 54 months, respectively. M refused antiretroviral therapy and died after 37^th^ months of infection. F started antiretroviral therapy after 38^th^ months of infection and continued to be followed to the 54^th^ months. Seven whole-blood and nine plasma samples were collected from F at nine time points, and five whole-blood and six plasma samples were collected from M at six time points (Table 1). Thus, in total, 12 immune cells from whole-blood and 15 plasma samples were studied.

### Laboratory Tests

To investigate the HIV-1 similarity and possible transmission between the couple, another six HIV-1-infected patients were enrolled as controls. The RNA was extracted from the plasma using the QIAamp MinElute Virus Spin Kit kit (QIAgen, Germany). The RNA was reverse-transcribed to generate complementary deoxyribonucleic acid (cDNA) using SuperScript III First-Strand Synthesis System by RT-PCR (Invitrogen, USA). The fragments of the HIV-1 *gag* gene region (HXB2 positions 781 to 1861) was selected for HIV-1 gene subtype analyses, as previously described^17^.

Total nucleic acids and RNA were extracted from whole-blood and plasma samples of HIV-1-infected couple, respectively. A target fragment corresponding to HXB2 positions 7170–7515 was amplified by nested PCR. The primers for the first round of amplification have been described elsewhere^18,27^. The primers used for the second round of amplification were X1-ATAAGKATAGGACCAGGACAA (HXB2 positions 7148 to 7168) and X2-ATGGGAGGRGCATACATTGCT (HXB2 positions 7541 to 7521). Sequencing was performed on the Miseq platform, with a 2 × 300 basepairs (bp) read length. Paired-end reads were assembled, and the raw tags were subjected to qualitative filtering, as previously described^28^. We adopted a conservative approach to ensure high sequencing quality: only HIV-1 quasispecies sequences appearing more than 50 times in whole-blood and plasma samples were analyzed^17,29,30^.

We used the HIV-1 Align program (http://www.HIV.Lanl.gov/content/sequence/VIRALIGN/viralign.html) to generate sequence alignments. Repetitive sequences were removed, resulting in a number of unique quasispecies sequences for each sample. We then calculated genetic distances and generated neighbor-joining trees with the Jukes–Cantor model, using MEGA 6.0.6 software. The trees generated were assessed by the bootstrap method, with 1000 replications.

## References

[1] Bu, D. & Tang, H. In *IEEE**International**Conference**on**Bioinformatics**and**Biomedicine*. 63–66 (2014).

[2] Palma AC (2007). Molecular epidemiology and prevalence of drug resistance-associated mutations in newly diagnosed HIV-1 patients in Portugal. Infection Genetics & Evolution Journal of Molecular Epidemiology & Evolutionary Genetics in Infectious Diseases.

[3] Cho YK, Jung Y, Lee JS, Foley BT (2012). Molecular evidence of HIV-1 transmission in 20 Korean individuals with haemophilia: phylogenetic analysis of the vif gene. Haemophilia.

[4] Katzenstein TL (1999). Nosocomial HIV-transmission in an outpatient clinic detected by epidemiological and phylogenetic analyses. AIDS.

[5] Ou CY (1992). Molecular epidemiology of HIV transmission in a dental practice. Science.

[6] Kaye M, Chibo D, Birch C (2009). Comparison of Bayesian and maximum-likelihood phylogenetic approaches in two legal cases involving accusations of transmission of HIV. Aids Research & Human Retroviruses.

[7] Lemey P (2005). Molecular testing of multiple HIV-1 transmissions in a criminal case. AIDS.

[8] Ison MG (2011). Transmission of human immunodeficiency virus and hepatitis C virus from an organ donor to four transplant recipients. American Journal of Transplantation.

[9] Rachinger A, Groeneveld PH, Van AS, Lemey P, Schuitemaker H (2011). Time-measured phylogenies of *gag*, *pol* and *env* sequence data reveal the direction and time interval of HIV-1 transmission. AIDS.

[10] Scaduto DI (2010). Source identification in two criminal cases using phylogenetic analysis of HIV-1 DNA sequences. Proceedings of the National Academy of Sciences of the United States of America.

[11] Salazargonzalez JF (2008). Deciphering Human Immunodeficiency Virus Type 1 Transmission and Early Envelope Diversification by Single-Genome Amplification and Sequencing. Journal of Virology.

[12] Metzker ML (2002). Molecular evidence of HIV-1 transmission in a criminal case. Proceedings of the National Academy of Sciences of the United States of America.

[13] Redd AD (2011). Identification of HIV Superinfection in Seroconcordant Couples in Rakai, Uganda, by Use of Next-Generation Deep Sequencing. Journal of Clinical Microbiology.

[14] Redd AD (2012). The Rates of HIV Superinfection and Primary HIV Incidence in a General Population in Rakai, Uganda. Journal of Infectious Diseases.

[15] Boutwell CL (2010). Todd M. Viral Evolution and Escape during Acute HIV‐1 Infection. The Journal of Infectious Diseases.

[16] Vandenhende MA (2014). Prevalence and evolution of low frequency HIV drug resistance mutations detected by ultra deep sequencing in patients experiencing first line antiretroviral therapy failure. Plos One.

[17] Zhao Q (2015). *Short Communication: Investigating a* Chain of HIV Transmission Events Due to Homosexual Exposure and Blood Transfusion Based on a Next Generation Sequencing Method. Aids Research & Human Retroviruses.

[18] Leitner T, Escanilla D, Franzen C, Uhlen M, Albert J (1996). Accurate reconstruction of a known HIV-1 transmission history by phylogenetic tree analysis. Proc Natl Acad Sci USA.

[19] Trkola A (2009). Inflammatory Genital Infections Mitigate a Severe Genetic Bottleneck in Heterosexual Transmission of Subtype A and C HIV-1. PLoS Pathogens.

[20] Kupfer B (2007). Fifteen years of Env C2V3C3 evolution in six individuals infected clonally with human immunodeficiency virus type 1. Journal of Medical Virology.

[21] Redd AD, Quinn TC, Tobian AAR (2013). Frequency and implications of HIV superinfection. The Lancet Infectious Diseases.

[22] Hallett TB (2013). HIV-1 Transmission during Early Infection in Men Who Have Sex with Men: A Phylodynamic Analysis. PLoS Medicine.

[23] Kraft CS (2012). Timing and source of subtype-C HIV-1 superinfection in the newly infected partner of Zambian couples with disparate viruses. Retrovirology.

[24] Liang C (2014). HIV-1 *pol* Diversity among Female Bar and Hotel Workers in Northern Tanzania. PLoS ONE.

[25] López, d. V. A., Tamamis, P., Kieslich, C. A. & Morikis, D. Insights into the Structure, Correlated Motions, and Electrostatic Properties of Two HIV-1 gp120 V3 Loops. *Plos One* **7**, e49925 (2012). 10.1371/journal.pone.0049925 PMC3501474 23185486

[26] Redd AD (2014). Rates of HIV-1 superinfection and primary HIV-1 infection are similar in female sex workers in Uganda. AIDS.

[27] Zhou Z (2011). Optimization of a low cost and broadly sensitive genotyping assay for HIV-1 drug resistance surveillance and monitoring in resource-limited settings. Plos One.

[28] Nixon DF (2010). Dynamics of HIV-1 Quasispecies during Antiviral Treatment Dissected Using Ultra-Deep Pyrosequencing. PLoS ONE.

[29] Kaidi F (2017). *Next generation sequencing-based* analysis of evolution of HIV-1 quasispecies in patients initiating antiretroviral therapy during acute infection. Chinese Journal of AIDS & STD.

[30] Yu-jie W, Yu-rong G, Yan J, Maofeng Q (2015). Transmission and evolution of HIV concordant heterosexual couples. Chinese Journal of AIDS & STD.

